# Development of the Gambling Disorder Identification Test (G-DIT): Protocol for a Delphi Method Study

**DOI:** 10.2196/12006

**Published:** 2019-01-08

**Authors:** Olof Molander, Rachel Volberg, Kristina Sundqvist, Peter Wennberg, Viktor Månsson, Anne H Berman

**Affiliations:** 1 Centre for Psychiatry Research Department of Clinical Neuroscience Karolinska Institutet Stockholm Sweden; 2 Stockholm Health Care Services Stockholm County Council Stockholm Sweden; 3 School of Public Health and Health Sciences University of Massachusetts Amherst, MA United States; 4 Department of Public Health Sciences Stockholm University Stockholm Sweden; 5 Stockholm Center for Dependency Disorders Stockholm Sweden

**Keywords:** consensus methods, Delphi technique, DSM-5, gambling, Gambling Disorder Identification Test, measurement, psychometrics, screening

## Abstract

**Background:**

Research on the identification and treatment of problem gambling has been characterized by a wide range of outcome measures and instruments. However, a single instrument measuring gambling behavior, severity, and specific deleterious effects is lacking.

**Objective:**

This protocol describes the development of the Gambling Disorder Identification Test (G-DIT), which is a 9- to 12-item multiple-choice scale with three domains: gambling consumption, symptom severity, and negative consequences. The scale is analogous to the widely used Alcohol Use Disorders Identification Test (AUDIT) and the Drug Use Disorders Identification Test (DUDIT).

**Methods:**

The G-DIT is developed in four steps: (1) identification of items eligible for the G-DIT from a pool of existing gambling measures; (2) presentation of items proposed for evaluation by invited expert researchers through an online Delphi process and subsequent consensus meetings; (3) pilot testing of a draft of the 9- to 12-item version in a small group of participants with problem gambling behavior (n=12); and (4) evaluation of the psychometric properties of the final G-DIT measure in relation to the existing instruments and self-reported criteria of the Diagnostic and Statistical Manual of Mental Disorders, 5th edition (DSM-5), among individuals with problem gambling and nonproblematic recreational gambling behaviors (n=600). This protocol article summarizes step 1 and describes steps 2 and 3 in detail.

**Results:**

As of October 2018, steps 1-3 are complete, and step 4 is underway.

**Conclusions:**

Implementation of this online Delphi study early in the psychometric development process will contribute to the face and construct validity of the G-DIT. We believe the G-DIT will be useful as a standard outcome measure in the field of problem gambling research and serve as a problem-identification tool in clinical settings.

**International Registered Report Identifier (IRRID):**

RR1-10.2196/12006

Protocol

## Introduction

### Overview

Gambling is the only addiction without any psychopharmacological substance use that has been recognized as a diagnosis by the American Psychiatric Association in the Diagnostic and Statistical Manual of Mental Disorders, 5th edition (DSM-5) [1]. Problem gambling is associated with poor mental and physical well-being in individuals with gambling problems [2]; in addition, their partners, parents, and children are negatively affected [3]. Problem gambling leads to severe negative consequences in important life domains such as finance, well-being, health, and relationships [1] and is associated with high rates of suicide ideation and attempts [4]. The clinical diagnostic criteria for pathological gambling were revised in 2013 and termed Gambling Disorder (GD) in the DSM-5 [1]. GD is part of the Substance-Related and Addictive Disorder category in DSM-5, in contrast to the Impulse Disorder category in DSM, 4th edition (DSM-IV) [5,6]. Other updates in the DSM-5 include removal of a previous criterion, illegal acts to finance gambling, and specification of disorder severity. Currently, fulfillment of 4-5 diagnostic criteria leads to a diagnosis of mild GD, 6-7 symptoms are diagnosed as moderate GD, and 8-9 symptoms are diagnosed as severe GD.

As a research field, problem gambling is still in its infancy and is 20-30 years behind research on substance use disorders [7]. Research on the identification and treatment of problem gambling has been characterized by a wide range of outcome measures and instruments [8], leading to difficulties in comparing the effectiveness of different treatments [9]. An additional current challenge for clinical assessment and research outcome measures is that only a few existing instruments have been validated using the relatively new DSM-5 diagnostic criteria for GD. Furthermore, measuring problem gambling from a treatment-oriented perspective is a challenge, as current screening instruments adopt a public health perspective and generally focus on consumption behaviors, symptoms, *or* negative consequences, but do not encompass all three domains.

To address the issue of variation in outcome measures, an expert panel of researchers convened in 2006 and agreed upon a set of characteristics that should define measures of problem gambling in future treatment studies; these characteristics are collectively known as the Banff consensus agreement [8]. Regarding the issue of including DSM-5 criteria in measures for identification of GD, researchers have proposed some specific DSM-5 criteria such as “chasing losses,” “repeated unsuccessful efforts to stop,” “tolerance,” “loss of control,” and “jeopardized/lost relationships/job” as important gambling measures, because they can be used from a psychometric perspective to better differentiate among various gambling groups as compared to the other GD diagnostic criteria [10-12].

In response to the Banff consensus agreement and the discussion regarding inclusion of specific DSM-5 criteria and with a goal of optimizing a treatment-oriented screening measure, our team is developing the Gambling Disorder Identification Test (G-DIT). We aim to establish a problem gambling-screening test analogous to the Alcohol Use Disorders Identification Test (AUDIT) [13] and the Drug Use Disorders Identification Test (DUDIT) [14]. Our test will include items in three domains: gambling consumption, symptom severity, and negative consequences. The development and validation of the G-DIT is part of the ongoing 6-year Responding to and Reducing Gambling Problems research program in Sweden.

The G-DIT is under development in four steps: (1) identification of items eligible for the G-DIT from a pool of existing gambling measures; (2) presentation of proposed items for evaluation by the authors of this article in a pilot Delphi round, followed by presentation of the proposed items for evaluation by a larger group of invited international expert researchers in a formal Delphi process, and finally, an international expert consensus meeting followed by additional smaller consensus meetings to resolve issues tabled at the international meeting; (3) pilot testing of a draft 9- to 12-item version in a small group of participants with problem gambling behavior (n=12); and (4) evaluation of psychometric properties of the final G-DIT measure in relation to existing instruments and self-reported DSM-5 criteria in individuals with problem gambling and nonproblematic recreational gambling behaviors (n=600). This article summarizes step 1 and describes steps 2 and 3 in detail; the results of steps 2 and 3 will be described in an upcoming publication, and an additional publication will detail step 4.

### Aims and Research Questions

The research questions are as follows:

- Which of the presented items should have the highest priority?
- What are the potential problems of the proposed G-DIT?
- How is the face validity of the G-DIT perceived?
- What psychometric findings could be of additional importance?

## Methods

### Study Approval and Consent

This study was approved by the Regional Ethics Board of Stockholm, Sweden (ref. no. 2017/1479-31). Approval was granted for the Delphi procedure and evaluation of the instrument in individuals with problem gambling behavior, individuals from gambling self-help groups, and individuals with recreational gambling behavior from a population sample. Informed consent was obtained from all stakeholders in the Delphi process as well as all participants with problem gambling behavior in the “think aloud” interviews. Participants were approached or volunteered via the methods outlined below. Individual Delphi stakeholders were sent a short email introducing the study, and more information on the study and consent forms were made available online. Individual responses were analyzed and presented anonymously in both the Delphi process and “think aloud” procedure. All participants provided consent for publication.

### Analysis of Existing Measures

In step 1, we aimed to identify the maximum number of existing gambling measures. We conducted an extensive literature search of review articles on gambling measures [15-17] and a prior unpublished collection of gambling measures compiled by local colleagues (A Nilsson and K Magnusson, personal communication, February 2017), which resulted in a list of 47 gambling measures (Table 1) [12,18-63]. Items from the measures were gathered in an item pool. Items with the same meaning were identified as doublets between instruments but classified as unique items within an instrument (eg, items in subscales). The final item pool consisted of 726 items, of which 583 were deemed unique items and 143 were deemed doublets; the latter were excluded from the item pool.

The first author categorized all items based on their content into four main categories and 27 subcategories: Gambling Consumption (Type of Game, Time Gambled, Sums, and Gambling Behavior); DSM-5 Criteria (Preoccupation, Tolerance, Loss of Control, Abstinence Symptoms, Escape, Chasing Losses, Lies, Social Consequences, and Relies on Other); Negative Consequences (General Problem, Health, Financial, Critique from Others, Illegal, and Other Negative Consequences); and Other (Motives for Gambling; Self-Efficacy; Situations or Relapse; Cognitive Distortions or Beliefs; Motivation; Anxiety, Depression, or Negative Effect; Alcohol or Drugs; and Other or Miscellaneous). The Other main item category was excluded, as it was not relevant to the G-DIT domains. Thereafter, three additional authors (blind to the original categorization) individually recategorized each item in the three remaining main categories (Gambling Consumption, DSM-5 Criteria, and Negative Consequences) and the predefined subcategories. Interrater reliability was calculated on the basis of the item-categorization agreement for all items, items per subcategory, and items per main category. Statistical analysis using Fleiss kappa [64] for 4 raters in R [65] showed that the interrater reliability ranged from fair to moderate (*k*=0.42 for all items and *k*=0.24, *k*=0.51, and *k*=0.51 for the relevant main item categories of Gambling Consumption, DSM-5 Criteria, and Negative Consequences, respectively).

### The Delphi Study

**Figure 1:**
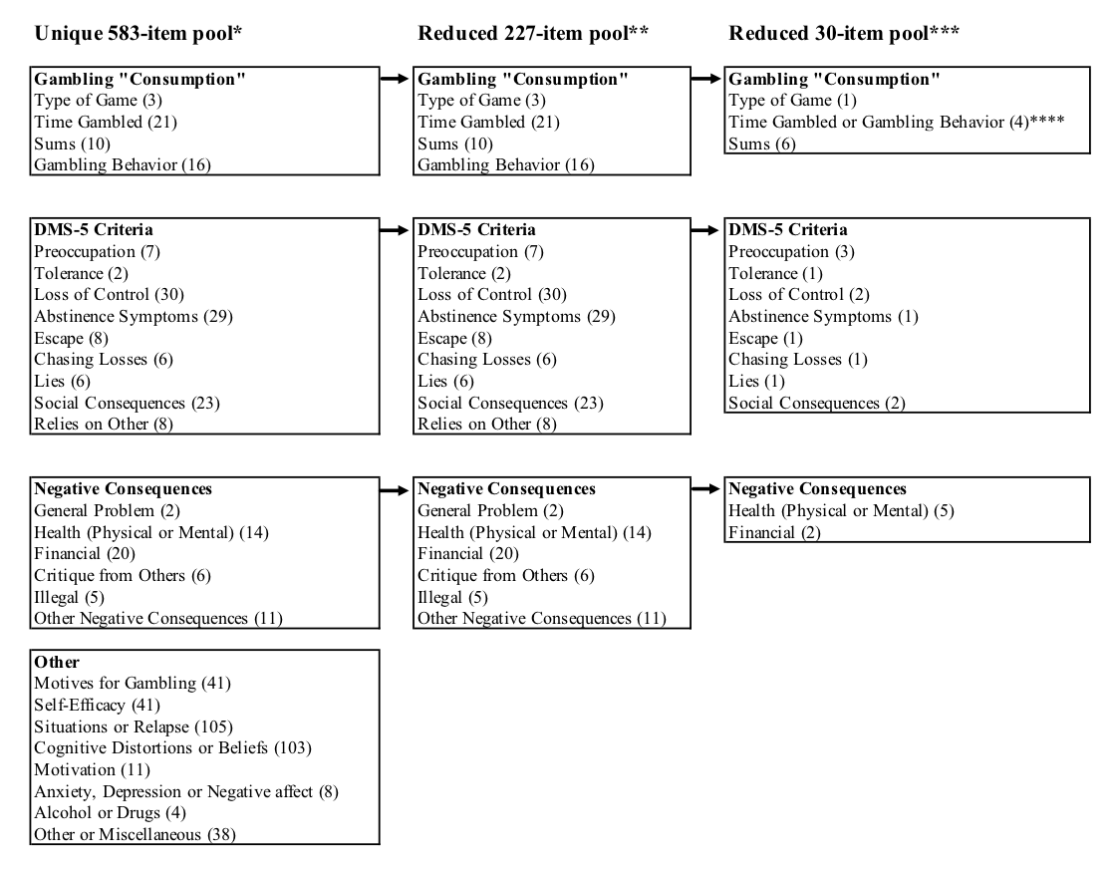
Item categorization and item selection for the Gambling Disorder Identification Test (G-DIT). The number of items is provided within parentheses. *Five items were lost in the initial categorization. **Interrater recategorization. ***Main Delphi. ****Time gambled and gambling behavior were merged to fit the G-DIT domains. DSM-5: Diagnostic and Statistical Manual of Mental Disorders, 5th edition.

We chose the Delphi method to collect feedback from expert researchers. The Delphi method is an iterative technique, comprising sequential questionnaires that are answered anonymously by many relevant stakeholders [66]. To prepare for the formal Delphi process in step 2, we conducted a pilot Delphi procedure in two rounds with the authors of the present study. In the preparation rounds, we evaluated 15 candidate items based on the interrater analysis in step 1. The criteria for selection were 75% agreement on the categorization and importance of these items. These two preparation rounds clarified the variation in expert evaluation of the items and led to a decision to increase the number of candidate items to 30 for the next formal Delphi rounds. The selection of these items was based on interrater agreement of items relevant to the G-DIT domains, previous psychometric findings regarding problem gambling, and the recommendations of the Banff consensus agreement [8]. An overview of the item categories is presented in Figure 1.

#### Panel Size and Recruitment

There are no accepted guidelines for the panel size in a Delphi analysis. Therefore, we determined our panel size on the basis of the practicality, scope, and time available, similar to previous studies [67,68]. Stakeholders were identified through contacts via our research group and team members of the ongoing research project “Responding to and Reducing Gambling Problems - Studies in Help-Seeking, Measurement, Comorbidity and Policy Impacts” (REGAPS) and through published research in the gambling field. We invited the following stakeholders to participate in the Delphi rounds and requested them to forward the invitation to other researchers in their network (snowball sampling): all authors of the Banff consensus [8] and previous psychometric research targeting specific DSM-5 symptoms [10-12]; presenters at the Alberta Gambling Research Institute’s 17th Annual Conference, 2018, which is the annual independent gambling conference in Banff (these individuals were identified as key influential gambling researchers for the international consensus meeting); all authors of reviews of gambling measures identified in our extended literature search [15-17]; corresponding and first and last authors of published articles or reports of the gambling measures identified in our extended literature search (Table 1); trial investigators including corresponding and first and last authors of reports of randomized trials in the field identified in published systematic reviews [9,69,70]; members of the REGAPS network; and members of the Gambling Research Network, which is a Swedish network for gambling research.

We addressed the potential for attrition between rounds through a personalized invitation, email reminders (every 5 days, but no more than two reminders in total), and provision of an easy interface, which minimized the time required to complete each round [67]. The Delphi-process questionnaire was uploaded on the online SurveyXact platform [71].

**Table 1:** Gambling measures (n=47) identified in the literature search.

Measure	Reference
The Brief Biosocial Gambling Screen	Gebauer et al, 2010 [41]
The Canadian Problem Gambling Index	Ferris et al, 2001 [23]
The Case-finding and Help Assessment Tool	Goodyear-Smith et al, 2008 [36]
The Consumption Screen for Problematic Gambling	Rockloff et al, 2012 [44]
The Control of Pathological Gambling Questionnaire	Saiz-Ruiz et al, 2005 [33]
The Cumulative Clinical Signs Method	Volberg et al, 1990 [55]
The Early Intervention Gambling Health Test	Sullivan, 2007 [50]
The Gamblers Self-Efficacy Questionnaire	May et al, 2003 [27]
The Gamblers’ Belief Questionnaire	Steenbergh et al, 2002 [56]
The Gambling Abstinence Self-Efficacy Scale	Hodgins et al, 2004 [29]
The Gambling Activity Measurement Tool	Jacksson et al, 2013 [57]
The Gambling Anonymous Twenty Questions	Toneatto et al, 2008 [53]
The Gambling Attitudes and Beliefs Survey	Breen et al, 1999 [49]
The Gambling Cognitions Inventory	McInnes et al, 2014 [58]
The Gambling Craving Scale	Young et al, 2009 [40]
The Gambling Follow-Up Scale	de Castro et al, 2005 [34]
The Gambling Motives Questionnaire	Stewart et al, 2008 [52]
The Gambling Motives Questionnaire Financial	Schellenberg et al, 2015 [47]
The Gambling Passion Scale	Rousseau et al, 2002 [25]
The Gambling Pathways Questionnaire	Nower et al, 2016 [48]
The Gambling Problem Index	Neighbors et al, 2002 [24]
The Gambling Quantity and Perceived Norms	Neighbors et al, 2002 [24]
The Gambling Readiness to Change Questionnaire	Raylu et al, 2004 [30]
The Gambling Refusal Self-Efficacy Questionnaire	Casey et al, 2008 [35]
The Gambling Symptom Assessment Scale	Kim et al, 2009 [38]
The Gambling Urge Scale	Raylu et al, 2004 [59]
The Gambling-Related Cognition Scale	Raylu et al, 2004 [30]
The Inventory of Gambling Situations	Turner et al, 2013 [45]
The Lie/Bet	Johnson et al, 1997 [21]
The Maroondah Assessment Profile for Problem Gambling^a^	Shek et al, 2009 [60]
The Massachusetts Gambling Screen	Shaffer et al, 1994 [19]
The NODS^b^-CLIP^c^	Volberg et al, 2011 [12]
The NODS-PERC^d^	Volberg et al, 2011 [12]
The NORC^e^ Diagnostic Screen for Gambling Problems	Gerstein et al, 1999 [22]
The NORC Diagnostic Screen for Gambling Problems Self-Administered	Gerstein et al, 1999 [22]
The Pathological Gambling Behavioral Self-Report Scale	Myrseth et al, 2011 [61]
The Problem and Pathological Gambling Measure	Willimas et al, 2013 [46]
The Problem Gamble Research and Treatment Centre Screen	—^f^
The Problem Gambling Severity Index	Ferris et al, 2001 [23]
The Scale of Gambling Choices	Baron et al, 1995 [20]
The South Oaks Gambling Screen	Lesieur et al, 1987 [18]
The South Oaks Gambling Screen Short	Room et al, 1999 [62]
The South Oaks Gambling Screen-Revised	Abbott et al, 1990 [63]
The Sydney Laval Gambling Scale	Blaszczynski et al, 2008 [51]
The Temptations for Gambling Questionnaire	Holub et al, 2005 [31]
The Victorian Gambling Screen	Tolchard et al, 2010 [42]
The Yale Brown Obsessive Compulsive Scale adapted for Pathological Gambling	Pallanti et al, 2005 [32]

^a^This measure was excluded from the item pool because it was not possible to obtain the instrument.

^b^NODS: National Opinion Research Center Diagnostic Screen for Gambling Problems.

^c^CLIP: Loss of Control, Lying, and Preoccupation.

^d^PERC: The National Opinion Research Center Diagnostic Screen for Gambling Problems - Preoccupation, Escape, Risked Relationships, and Chasing.

^e^NORC: National Opinion Research Center.

^f^Published reference not found.

#### The Delphi Questionnaire and Rounds

Stakeholders were instructed to log on to the online questionnaire where they first read information about the study and electronically signed an informed consent form and to provide data on demographic characteristics including gender, country, number of years engaged in gambling-related work, and profession. Thereafter, the stakeholders viewed the proposed items in the measure. The items were listed randomly to avoid assigning any order of importance to the items. For each item, the stakeholders were instructed to provide feedback on the psychometric relevance and accuracy, semantic structure, and multiple-choice alternatives. In addition, the stakeholders were asked to rate each item on a scale of 1-9, where scores of 1-3 were considered “not important for inclusion,” 4-6 were considered “important but not critical,” and 7-9 were considered “critical for inclusion.” Further, an open-text field was provided with each item, through which the stakeholders could provide additional feedback or information; for example, important psychometric findings that were previously not noted by our research group. A rationale for each item shown from a psychometric perspective was presented; for example, “Item 5. How often do you gamble to win back money you lost? Never, Less than monthly, Monthly, Weekly, or Daily or almost daily.” The rationale for inclusion of this item is that “Chasing losses” is a key symptom in the diagnostic criteria of GD. A recent latent class analysis of data found that “the main diagnostic item serving to discriminate recreational from problem gamblers was endorsement of ‘chasing losses’” [10].

The Delphi survey was repeated in a second round. The importance of completing both rounds was emphasized to the stakeholders in the study information. After completion of Round 1, all stakeholders were invited to Round 2, where they were asked to respond to the questionnaire again. In addition to the previously described content, the stakeholders were presented with an anonymous summary of the other stakeholders’ responses. Using this information, each expert was asked to reflect on their own rating in relation to the overall group rating and rate each item again. After Round 2, the results of the Delphi analysis were summarized.

#### Consensus Meeting

After the end of the Delphi rounds, a consensus meeting was held with a subgroup of international researchers attending the Alberta Gambling Research Institute’s 17th Annual Conference. The results from the Delphi were presented and discussed, and a consensus was reached to determine the final G-DIT item structure. To review the results and adjust the G-DIT measure accordingly, subsequent consensus meetings were held on issues tabled at the international consensus meeting. Participants at these meetings were the authors of the present article and two Swedish participants of the international consensus meeting. At the end of the consensus process, the G-DIT was also translated into Swedish using a back-translation procedure [72].

### Think Aloud Procedure

Swedish individuals (n=12) with problem gambling behavior were recruited from treatment-seeking and self-help groups. The inclusion criteria were willingness to participate in the study and personal experience of gambling problems. The participants provided feedback according to the “think aloud” procedure [73,74]. They were instructed in advance to think aloud “as if alone in the room.” First, the participants practiced the procedure when presented with an instruction text. Subsequently, they were presented with each item in the draft version of the Swedish G-DIT. Their comments were noted by the interviewer, who otherwise did not intervene, except to provide reminders to think aloud. The results of the interviews were analyzed using content analysis. Thereafter, the G-DIT was adjusted further to increase face validity of the measure.

### Psychometric Evaluation in Treatment-Seeking and Population Cohorts

In the final step of the study protocol, the psychometric properties of the G-DIT will be evaluated in relation to the DSM-5 diagnostic criteria for GD [1] and other gambling instruments through survey data and clinical interviews. Data will be collected from treatment-seeking and self-help group samples as well as population samples including people with recreational gambling behavior in Sweden (n=600). The inclusion criteria for treatment-seeking and self-help group participants will be a total score of ≥3 on the Problem Gambling Severity Index (PGSI) [23], 18-85 years of age, ability to read and write Swedish, and not fulfilling the criteria for a manic episode. The inclusion criteria for the population sample will be 18-85 years of age and the ability to read and write Swedish. The procedure will first be piloted with a cohort of participants seeking treatment for problem gambling (n=80), after which additional adjustment of the G-DIT, such as further reduction of items, may be performed.

## Results

Funding sources for the G-DIT project include the Swedish Research Council for Health, Working Life and Welfare (Grant no. 2016-07091), covering a 6-year program grant entitled REGAPS, and development funds from the Stockholm Health Care Services, Stockholm County Council, for identification and treatment of problem gambling. As of November 2018, steps 1-3 have been completed, and step 4 is underway.

## Discussion

This article describes a study protocol to develop a new measure for the assessment of problem gambling. We describe methods for item generation, instrument development, and procedures for testing the face and construct validity by collecting feedback from expert researchers and participants with problem gambling behavior. This study will set the foundation for a subsequent psychometric study that will aim to evaluate the psychometric properties of the G-DIT in relation to existing instruments, clinical interviews, and self-reported DSM-5 criteria among Swedish individuals with problem gambling behavior from treatment-seeking and self-help groups samples as well as population samples including people with recreational gambling behaviors.

This study protocol has several strengths. First, our extensive literature search identified a large number of existing gambling measures. Our overview indicated that no single existing measure seemed to adequately fulfill the recommendations of the Banff consensus. Second, only a few measures have been validated by the DSM-5 diagnostic criteria for GD. Third, many existing measures include item responses with generalized multiple or dichotomous “yes” or “no” response options rather than specific behavior or time frequencies. Fourth, the use of digital platforms in this study facilitates broad national and international collaborations in emerging research fields such as problem gambling. Our scope for recruiting expert researchers was wide. Implementation of a Delphi study early in the psychometric development process will contribute to the face and construct validity of the final measure. Through the Delphi process, several key problematic issues for measuring gambling-related content were identified and will be discussed in the forthcoming publication. Our systematic procedure will contribute to the establishment of public health guidelines for gambling behavior, similar to the guidelines for alcohol consumption currently available in many countries.

The final G-DIT will consist of three domains: gambling consumption, symptom severity, and negative consequences. In addition, an appendix on expenditure and gambling types will be included. We believe the G-DIT will complement existing screening scales in upcoming intervention trials among community and treatment-seeking groups and prove useful as a standard outcome measure for change in problem gambling behavior. An additional potential area of use is the identification of problem gambling in clinical settings.

## Abbreviations

CLIP: Loss of Control, Lying, and Preoccupation
DSM-5: Diagnostic and Statistical Manual of Mental Disorders, 5th edition
DSM-IV: Diagnostic and Statistical Manual of Mental Disorders, 4th edition
G-DIT: Gambling Disorder Identification Test
NODS: National Opinion Research Center Diagnostic Screen for Gambling Problems
NORC: National Opinion Research Center
PERC: The National Opinion Research Center Diagnostic Screen for Gambling Problems - Preoccupation, Escape, Risked Relationships, and Chasing
REGAPS: Responding to and Reducing Gambling Problems - Studies in Help-Seeking, Measurement, Comorbidity, and Policy Impacts
